# Biomarkers for Monitoring of Changes in Disease Activity in Ulcerative Colitis

**DOI:** 10.3390/jcm12227165

**Published:** 2023-11-18

**Authors:** Yoshihiro Tatsumi, Kazuki Kakimoto, Azusa Hara, Noboru Mizuta, Keijiro Numa, Naohiko Kinoshita, Kei Nakazawa, Ryoji Koshiba, Yuki Hirata, Kazuhiro Ota, Takako Miyazaki, Shiro Nakamura, Kayoko Sakagami, Shoko Arimitsu, Hiroaki Ito, Hiroki Nishikawa

**Affiliations:** 1 2nd Department of Internal Medicine, Osaka Medical and Pharmaceutical University, Takatsuki City 569-8686, Japan; t_cop_doradora@yahoo.co.jp (Y.T.); wakayamakisyu@yahoo.co.jp (A.H.); noboru.mizuta@ompu.ac.jp (N.M.); jeni_like_0902@yahoo.co.jp (K.N.); naohiko.kinoshita@ompu.ac.jp (N.K.); kei.nakazawa@ompu.ac.jp (K.N.); ryouji.koshiba@ompu.ac.jp (R.K.); yuki.hirata.ln@ompu.ac.jp (Y.H.); clash_kaz@yahoo.co.jp (K.O.); takako.miyazaki@ompu.ac.jp (T.M.); saab460@gmail.com (S.N.); hiroki.nishikawa@ompu.ac.jp (H.N.); 2 Kinshukai Infusion Clinic, Osaka-shi 530-0011, Japan; office@kic-clinic.jp (K.S.); aripikari@ares.eonet.ne.jp (S.A.); lovemagicflute@yahoo.co.jp (H.I.)

**Keywords:** ulcerative colitis, biomarkers, LRG (leucine-rich alpha-2-glycoprotein), fecal calprotectin, anti-TNF antibody therapy

## Abstract

Background: In recent years, various biomarkers of ulcerative colitis (UC) have emerged; however, few studies have simultaneously examined the utility of multiple biomarkers for monitoring disease activity. Additionally, serum leucine-rich alpha-2 glycoprotein (LRG), a new biomarker, may show a blunt response to anti-TNF antibody therapy. This prospective study explored effective biomarkers that could monitor disease activity changes in patients with UC. In addition, we examined the effect of anti-TNF antibody therapy on changes in LRG. Methods: Blood and stool samples were collected twice from patients with UC: at baseline and at least 8 weeks later. Changes in serum LRG, interleukin (IL)-6, prealbumin (pre-Alb), high-sensitivity C-reactive protein (hs-CRP), CRP, and fecal calprotectin (FC) were measured and correlated with changes in disease activity. The relationship between anti-TNF antibody therapy and LRG levels was also examined in patients with the same disease activity. Results: Forty-eight patients with UC (96 samples) were analyzed. ΔLRG and ΔIL-6 correlated strongly with the change in the partial Mayo (pMayo) score between the two time points (ΔpMayo) (r = 0.686, 0.635, respectively). In contrast, FC and IL-6 were particularly accurate predictors of clinical remission, and their area under the curves (AUCs) were significantly higher than that of CRP (AUC: 0.81, 0.76 vs. 0.50; *p* = 0.001, 0.005). No association was found between the administration of anti-TNF antibody preparations and the LRG values. Conclusions: Correlations were found between changes in UC disease activity and LRG, IL-6, pre-Alb, hs-CRP, CRP, and FC. LRG reflects disease activity during anti-TNF antibody therapy.

## 1. Introduction

Ulcerative colitis (UC) is a chronic inflammatory bowel disease of unknown etiology that affects the large intestine. In recent years, various biomarkers of UC have emerged, making it possible to objectively evaluate disease activity. In the clinical setting of UC, the measurement of biomarkers has various implications, including estimation of the grade and severity of mucosal inflammation, measurement of response to therapy, and surveillance of relapse after induced remission [1]. It is known that individual variations exist in biomarker levels, even within groups of patients with the same level of disease activity [2]. Therefore, aside from measuring biomarker levels at baseline and during treatment, it is also important to measure the changes of these biomarkers during the clinical course of the disease [2]; this is especially valuable when monitoring for relapse and treatment response. Even so, no prospective studies have been conducted to simultaneously track multiple biomarkers in relation to changes in disease activity. It is clinically important to understand the correlation of disease activity with biomarkers in monitoring the changes in disease activity; however, such information is currently lacking. For example, the fecal calprotectin (FC) is an excellent biomarker for determining mucosal healing; however, it has large numerical variability and may not accurately reflect treatment-induced changes in disease activity [3,4].

Various biomarkers have been reported to correlate with disease activity in UC. Calprotectin is an inflammation-associated protein that is primarily localized within neutrophilic cytoplasm, and its presence in stool indicates neutrophil migration into the gastrointestinal tract during inflammation [5,6]. FC can predict endoscopic activity with high sensitivity [7]. However, it is challenging to utilize this biomarker as a measure of treatment response since the change in FC levels after treatment initiation is highly variable between individuals [8,9]. Leucine-rich alpha-2 glycoprotein (LRG) is a plasma glycoprotein containing a repeating sequence of leucine-rich motifs [10]. Inflammatory cytokines, such as tumor necrosis factor (TNF)-α, interleukin (IL)-1β, and IL-6, induce LRG production in the hepatocytes, neutrophils, and macrophages [11,12]. In recent years, LRG has attracted considerable attention because of their marked correlation with disease activity in inflammatory bowel disease (IBD), particularly in UC [13]. IL-6 is a multifaceted mediator that modulates the intestinal immune system through classical IL-6 signaling or IL-6 trans-signaling [14,15]. Serum IL-6 levels are markedly elevated in patients with active IBD and have been shown to be positively correlated with disease activity [16,17]. C-reactive protein (CRP) is an acute-phase protein produced by hepatocytes upon IL-6 stimulation; it is also the most well-studied inflammatory parameter in patients with IBD [7,18]. However, the sensitivity of CRP to endoscopic activity in patients with IBD is low [8]. CRP levels are also associated with high-sensitivity CRP (hs-CRP) and prealbumin (pre-Alb) levels. The hs-CRP assay can measure values below the detection limit of CRP, and its results are correlated with disease activity in patients with UC [19,20]. Furthermore, pre-Alb levels are inversely correlated with disease activity in patients with IBD [21,22]. Although studies have reported various useful biomarkers in UC, few have simultaneously analyzed their correlation with disease activity.

Another factor that has to be taken into consideration is the interaction between biomarkers. For example, CRP is less likely to be elevated under anti-IL-6 receptor antibody therapy in patients with rheumatoid arthritis since the release of CRP is affected by IL-6; therefore, it is not useful for assessing disease activity [23]. Similarly, LRG is induced by stimuli such as TNFα [12]; therefore, under the administration of anti-TNFα antibody preparations, which are typical biologics for the treatment of refractory UC, the reactivity of LRG may be slowed down with a decrease in TNFα. However, there have been no reports on LRG reactivity in patients with UC receiving anti-TNFα antibody agents.

This prospective study aimed to investigate the correlation of six biomarkers (FC, serum LRG, IL-6, pre-Alb, hs-CRP, and CRP) with the changes in disease activity in patients with UC. As a secondary endpoint, we also examined whether serum LRG accurately reflects disease activity in patients with UC receiving anti-TNFα antibody preparations.

## 2. Materials and Methods

### 2.1. Patients

Patients attending the Kinshukai Infusion Clinic between May 2020 and April 2021 were prospectively enrolled. The diagnosis of UC was based on a combination of clinical presentation, endoscopic findings, histology, and the exclusion of alternative diagnoses. Biomarker measurements were carried out at two time points; once at baseline and once at least 8 weeks after the first examination. At each measurement, patients provided blood and stool samples at the same time. LRG, IL-6, pre-Alb, hs-CRP, and CRP levels were measured in two blood samples, and FC was measured using two stool samples from the same patient. Demographic data, current medications, clinical disease activity, and laboratory blood data were recorded at the two time points when the samples were collected. Partial Mayo (pMayo) scores were used to assess clinical disease activity, excluding the endoscopic subscores [24]. Clinical remission was defined as a pMayo score ≤2 with each subscore ≤1.

### 2.2. Study Endpoints

The primary endpoint was the correlation between the change in clinical activity and the change in each biomarker at the two time points. The main secondary endpoint was the diagnostic accuracy of each biomarker for clinical remission. Another secondary endpoint was the change in serum LRG reactivity with anti-TNF antibody therapy. We compared LRG values between patients with and without anti-TNF antibody therapy who had the same level of disease activity.

### 2.3. Biomarker Measurements

Serum LRG, IL-6, pre-Alb, hs-CRP, and FC levels were analyzed at the laboratory of LSI MEDIENCE Co., Ltd., Osaka, Japan. Serum LRG levels were measured using a NANOPIA LRG kit based on the latex turbidimetric method (SEKISUI MEDICAL Co., Ltd., Tokyo, Japan). Serum IL-6 levels were measured by chemiluminescent enzyme immunoassay (CLEIA) using the Quanti Glo Human IL-6 Immunoassay kit (R&D Systems Inc., Minneapolis, MN, USA). Serum pre-Alb levels were measured by a turbidimetric immunoassay (TIA) using the N-assay TIA Prealbumin Nittobo (NITTOBO MEDICAL Co., Ltd., Tokyo, Japan). Serum hs-CRP levels were measured using nephelometry (N-latex CRPII; Siemens Healthineers, Osaka, Japan). FC was measured by fluorescence enzyme immunoassay (FEIA) using Elia Calprotectin 2 (Thermo Fisher Scientific, Tokyo, Japan). Serum CRP levels were analyzed by an in-hospital laboratory using CHM-4120 (NIHON KOHDEN Co., Tokyo, Japan).

### 2.4. Statistical Analysis

Quantitative data were summarized using medians and interquartile ranges (IQR), while categorical variables were presented as frequencies and percentages. We used the Wilcoxon signed-rank test to compare non-parametric paired values. To evaluate the predictive performance of each biomarker for clinical remission, the receiver operating characteristic (ROC) curves were plotted to calculate the area under the ROC curve. Pearson’s test was performed to analyze the correlation between the biomarkers and activity indices. Statistical significance was set as *p* < 0.05 (two-sided test). The sample size was based on previous studies where the correlation between biomarkers and pMayo scores were assessed [25]. Using a two-sided hypothesis with α = 0.05, we estimated that 46 patients would be required, providing 80% power to detect a moderate correlation (r = 0.4) between biomarkers and pMayo scores [26]. To be conservative, we planned to enroll 48 patients in case of protocol violations or technical difficulties associated with blood sampling. All statistical analyses were performed using JMP^®^, Version 15.2.1, SAS Institute, Inc., Cary, NC, 1989-2021, USA.

## 3. Results

### 3.1. Patients’ Characteristics

There were 48 patients (24 men and 24 women) with a median age of 43.5 years, a median disease duration of 12.5 years, and an extent of disease as follows: 30 cases of total colitis type and 18 cases of left-sided colon type (Table 1). At the first measurement, treatments included the use of 5ASA preparations in 87.5% of patients, corticosteroids in 2.1%, azathioprine in 14.6%, and molecularly targeted drugs in 52.1%. At the second measurement, patients were receiving the same treatments, with the exception of molecularly targeted drugs, which were used in 50% of patients (vs. 52.1% at the previous measurement). The median pMayo was one (0–3) at the first measurement and one (0–2.3) at the second measurement. Clinical remission was observed in 35 patients (72.9%) at the first measurement and 36 patients (75%) at the second measurement. The median interval between the two measurements was 60.5 (56–82.5) days.

### 3.2. Comparison of Various Biomarkers in Clinical Remission and Non-Remission

**Figure 1:**
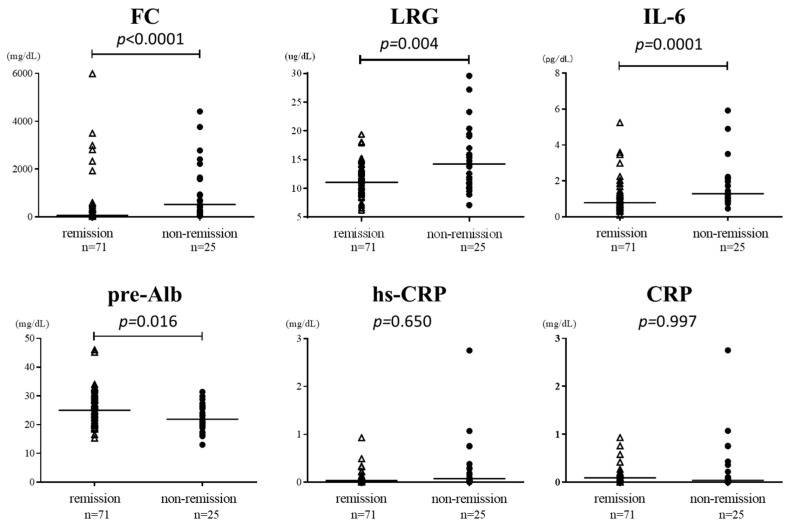
Comparison of each biomarker in patients in clinical remission and those not in remission. FC, fecal calprotectin; LRG, leucine-rich alpha-2 glycoprotein; IL-6, interleukin-6; pre-Alb, prealbumin; hs-CRP, high-sensitivity C-reactive protein.

A total of 96 samples were used to compare the levels of each biomarker in patients with clinical remission and non-remission of UC. As shown in Figure 1, three biomarkers had significantly higher medians in patients with active disease than those in remission: FC (522 [224–1650] vs. 65.8 [24.6–228.5] μg/g, respectively), LRG (14.2 [10.9–17] vs. 11.1 [9.5–13.4] μg/mL, respectively), and IL-6 (1.29 [1.02–2.09] vs. 0.79 [0.59–1.16] pg/dL). In contrast, pre-Alb had a significantly lower median in patients with active disease (21.9 [19.5–26] vs. 25 [21.9–29] mg/dL, respectively). The two remaining biomarkers were not statistically different between patients with active disease and those with remission: CRP (0.04 [0–0.22] vs. 0.09 [0–0.11] mg/dL, respectively) and hs-CRP (0.076 [0.023–0.283] vs. 0.035 [0.013–0.087] mg/dL, respectively). 

### 3.3. Diagnostic Accuracy of Each Biomarker for Clinical Remission

**Figure 2:**
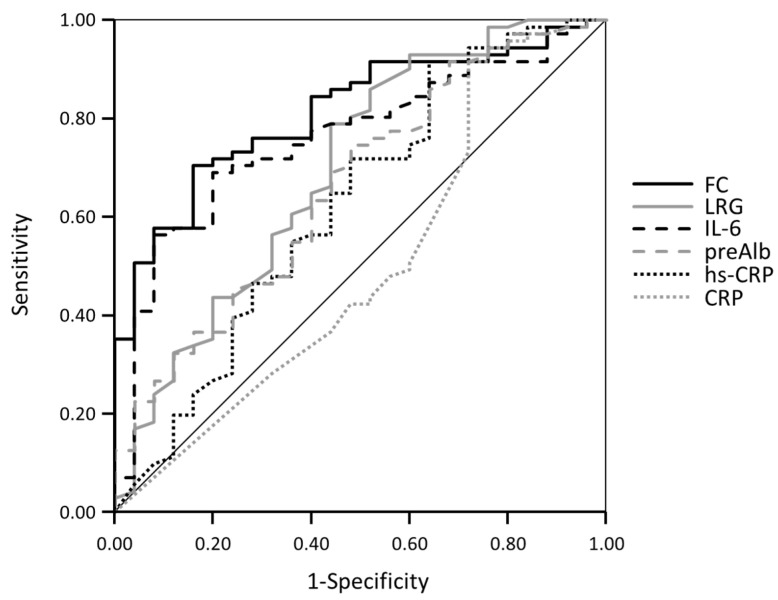
Receiver operating characteristic curve of the respective biomarkers for predicting clinical remission. FC, fecal calprotectin; LRG, leucine-rich alpha-2 glycoprotein; IL-6, interleukin-6; pre-Alb, prealbumin; hs-CRP, high-sensitivity C-reactive protein.

Subsequently, the diagnostic accuracy of each biomarker for the clinical remission of UC was examined by calculating the optimal cutoff value, sensitivity, and specificity of each biomarker using ROC curves and comparing the area under the curve (AUC). The cut-off value of FC was 184 μg/g, with a sensitivity of 84.0% and specificity of 70.4%, while that of LRG was 13.8 μg/mL, with a sensitivity of 56.0% and specificity of 78.9% (Figure 2).

Only the AUCs for FC and IL-6 were significantly higher than the AUC for CRP. (AUC:0.81, 0.76 vs. 0.50; *p* = 0.001, 0.005) (Table 2). In contrast, LRG had moderate accuracy (AUC = 0.70), which was higher than the AUC of CRP but not significantly (*p* = 0.105). The AUCs for pre-Alb and hs-CRP were low and did not differ significantly from those for CRP (AUC:0.66, 0.63 vs. 0.50; *p* = 0.141, 0.333).

### 3.4. Correlation between Clinical Activity and Each Biomarker

We examined the correlation between the clinical activity of UC and each biomarker using a total of 96 samples (Table 3) and found that LRG and IL-6 were significantly correlated with pMayo (r = 0.442 and 0.405, respectively). Levels of hs-CRP, FC, CRP and pre-Alb showed relatively weak correlations (r = 0.361, 0.354, 0.310, and −0.231, respectively).

### 3.5. Correlation between Changes in pMayo Scores at Two Time Points and Change in Each Biomarker

**Figure 3:**
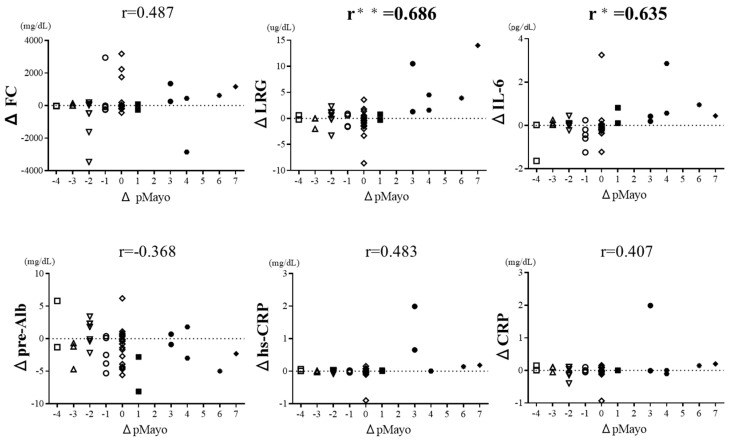
Correlation between pMayo change and change in each biomarker at two time points. When the correlation coefficient of ΔCRP was compared with those of the other biomarkers, those of ΔLRG and ΔIL-6 were significantly higher than that of ΔCRP: ** *p* < 0.01, * *p* < 0.05. FC, fecal calprotectin; LRG, leucine-rich alpha-2 glycoprotein; IL-6, interleukin-6; pre-Alb, prealbumin; hs-CRP, high-sensitivity C-reactive protein. The shape of the symbols in the graphs is represented by a different shape for each ΔpMayo value.

We examined the correlation between changes in clinical activity and changes in each biomarker at two time points in the same patients (Figure 3). The pMayo at the second measurement was lower than that at the first measurement in 12 patients, higher in 8 patients, and unchanged in 28 patients. ΔLRG and ΔIL-6 correlated strongly with the change in pMayo (ΔpMayo) (r = 0.686, *p* < 0.0001 and 0.635, *p* < 0.0001, respectively). ΔFC, Δpre-Alb, Δhs-CRP, and ΔCRP were also correlated with ΔpMayo but not strongly (r = 0.487, −0.368, 0.483, and 0.407, respectively; *p* < 0.01). When the correlation coefficient of ΔCRP was compared with those of the other biomarkers, those of ΔLRG and ΔIL-6 were significantly higher than that of ΔCRP (*p* = 0.005, 0.029). In contrast, the correlation coefficients of ΔFC, Δpre-Alb, and Δhs-CRP were not significantly different from that of ΔCRP (*p* = 0.497, 0.787, and 0.497, respectively). These results suggest that LRG and IL-6 in particular are useful biomarkers for the sensitive detection of changes in disease activity in UC.

### 3.6. Association between Anti-TNF Antibody Preparations and LRG

**Figure 4:**
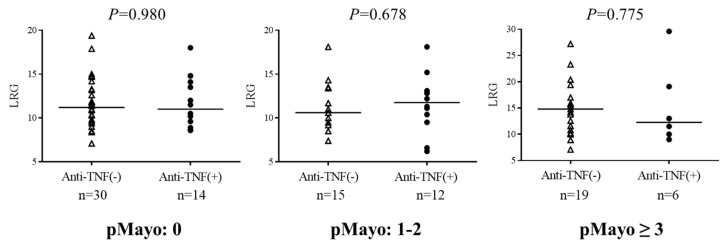
Comparison of LRG values between patients receiving anti-TNF antibodies and those not receiving anti-TNF antibodies according to the grade of clinical disease activity. LRG, leucine-rich alpha-2 glycoprotein; pMayo, partial Mayo score.

To examine whether LRG reactivity was blunted in patients with UC receiving anti-TNF antibody preparations, we compared LRG values between patients with and without anti-TNF antibody preparations; this comparison was only made between patients with the same clinical disease activity (pMayo: 0, 1–2, and ≥3). No association was observed between LRG and the administration of anti-TNF antibody preparations in any of the disease activity groups (Figure 4).

In addition, the correlation coefficients between LRG and other biomarkers were examined to determine whether the presence or absence of anti-TNF agents was associated with LRG (Table 4). pMayo and FC did not change with anti-TNF antibody administration (pMayo; anti-TNF (−): 0.470 vs. anti-TNF (+): 0.393, *p* = 0.66, FC; anti-TNF (−): 0.380 vs. anti-TNF (+): 0.384, *p* = 1). These results suggested that the administration of anti-TNF antibody preparations had no apparent effect on LRG reactivity. In contrast, the correlation coefficient between hs-CRP and LRG was significantly higher in patients receiving anti-TNF agents than in those not receiving anti-TNF agents (hs-CRP; anti-TNF (−): 0.593 vs. anti-TNF (+): 0.825, *p* = 0.034). The correlation coefficient between CRP and LRG also tended to be higher in patients receiving anti-TNF antibody agents (CRP; anti-TNF (−):0.620 vs. anti-TNF (+):0.827, *p* = 0.057).

## 4. Discussion

In this study, we searched for biomarkers that could accurately monitor changes in disease activity in UC. When six biomarkers were prospectively and simultaneously assessed, LRG and IL-6 were particularly strongly associated with changes in disease activity. The correlation with changes in disease was stronger for these two biomarkers compared with CRP. In recent years, the value of using biomarkers to objectively assess disease activity in inflammatory bowel disease has gained recognition. LRG is a biomarker that has recently received attention and has the advantage of being easily measurable in serum [14]. This is the first prospective study to simultaneously analyze the sensitivity of various biomarkers including LRG to changes in disease activity. The results of this study suggest that LRG levels acutely reflect changes in disease activity.

It has been reported that FC values tend to correlate positively with endoscopic inflammation in patients with clinically remitted UC and are considered useful for monitoring relapse during clinical remission because of their high sensitivity to microinflammation [8,27,28,29]. The present study suggested that FC was able to distinguish clinical remission and non-remission very accurately; however, it was not very sensitive to changes in disease activity. Therefore, different biomarkers should be utilized to serve different clinical purposes. 

Certain biomarkers may be undetectable in patients treated with biological agents. It is known that serum CRP, which is induced by IL-6, is less likely to be elevated in patients with rheumatoid arthritis who are being treated with anti-IL-6 receptor antibody preparations [23]. Similarly, since LRG is induced by stimulation from inflammatory cytokines such as TNFα, IL-1β, and IL-6, this study examined whether LRG is less likely to be detected in patients with UC receiving anti-TNF antibody preparations. No particular change in LRG reactivity was observed in these patients. These results suggest that LRG is a useful marker of disease activity even in patients receiving anti-TNF antibody preparations.

Interestingly, LRG was strongly correlated with CRP and hs-CRP, and the correlation was even stronger in patients receiving anti-TNF antibody preparations. Since the expression of TNFα is presumably reduced in patients treated with anti-TNF antibodies, and the correlation between IL-6 and LRG was very weak, these two cytokines (TNFα was and IL-6) should have a low effect on LRG. IL-6 is the major proinflammatory cytokine that induces CRP, although IL-1β is also involved [30,31]. These findings suggest that the reason for the strong correlation between LRG and CRP levels is that IL-1β is the main inflammatory cytokine that induces LRG following anti-TNF antibody treatment. 

This study has a few limitations. First, colonoscopy was not performed, thus, the association between endoscopic activity and each biomarker could not be examined. One of the major benefits of biomarkers in UC is that they can be used to estimate mucosal inflammation without colonoscopy. Clinical disease activity cannot accurately reflect the disease state on its own; however, since mucosal inflammation was not assessed in this study, only clinical disease activity was taken into consideration when assessing biomarkers. Second, due to the small number of cases, it was not possible to compare the diagnostic accuracy between biomarkers and investigate which biomarkers are more useful. Third, of the 48 patients enrolled, pMayo changed in only 20 patients. The presence of many unchanged cases may have weakened the correlation analyses; however, we also included the unchanged cases in the analysis, bearing in mind that the biomarker values varied slightly, even in cases where pMayo did not change. Fourth, this was a cross-sectional study of patients with UC in an outpatient setting, and patients with active disease who required hospitalization were not enrolled. Fifth, the study cohort consisted of patients regardless of their treatment timing. Sixth, biomarkers are expensive to measure, and their use in routine practice may be limited. 

## 5. Conclusions

Correlations were found between changes in UC disease activity and LRG, IL-6, pre-Alb, hs-CRP, CRP, and FC. The LRG reflects disease activity in patients with UC receiving anti-TNF antibody agents.

## Figures and Tables

**Table 1:** Baseline Demographics and Clinical Characteristics.

Number of Patients	*n* = 48
Male/Female, *n*	24/24
Age, year, median (IQR)	43.5 (32.8–52.3)
Duration of disease, year, median (IQR)	12.5 (7–18.3)
UC location; Left side/Extensive, *n*	30/18
Medications for UC	
Aminosalicylates, *n* (%)	42 (87.5)
Azathioprine, *n* (%)	7 (14.6)
Corticosteroids, *n* (%)	1 (2.1)
Anti-TNF-α agents, *n* (%)	17 (35.4%)
(infliximab, adalimumab, golimumab, *n* (%))	(10/4/3 (20.8/8.3/6.3))
Vedolizumab, *n* (%)	4(8.3)
Ustekinumab, *n* (%)	1(2.1)
Tofacitinib, *n* (%)	2(4.2)
Partial Mayo score, median (IQR)	1 (0–3)
Clinical remission/non-remission	35/13
WBC, /μL, median (IQR)	6300 (5050–7300)
Hb, g/dL, median (IQR)	13.8 (13.0–15.5)
Albumin, g/dL, median (IQR)	4.5 (4.3–4.6)
CRP, mg/L, median (IQR)	0.05 (0–0.14)

IQR, interquartile range; UC, ulcerative colitis; WBC, white blood cell; Hb, hemoglobin, CRP, C-reactive protein.

**Table 2:** Analysis by receiver operating characteristic curve of the respective biomarkers for clinical remission (*n* = 96).

Variables	AUC (95%CI)	*p*-Value (vs. CRP)
CRP	0.50 (0.36–0.64)	
FC	0.81 (0.72–0.90)	0.001
IL-6	0.76 (0.66–0.86)	0.005
LRG	0.70 (0.57–0.83)	0.105
pre-Alb	0.66 (0.54–0.79)	0.141
hs-CRP	0.63 (0.49–0.76)	0.333

AUC, area under the curve; CRP, C-reactive protein; FC, fecal calprotectin; IL-6, interleukin-6; LRG, leucine-rich alpha-2 glycoprotein; pre-Alb, prealbumin; hs-CRP, high-sensitivity CRP.

**Table 3:** Correlation between clinical activity and each biomarker (*n* = 96).

Variables	*r*	*p*
FC	0.354	0.0004
LRG	0.442	<0.0001
IL-6	0.405	<0.0001
pre-Alb	−0.231	0.0238
hs-CRP	0.361	0.0003
CRP	0.310	0.0021

FC, fecal calprotectin; LRG, leucine-rich alpha-2 glycoprotein; IL-6, interleukin-6; pre-Alb, prealbumin; hs-CRP, high-sensitivity C-reactive protein.

**Table 4:** The correlation coefficients between LRG and other biomarkers with and without anti-TNF antibody preparations.

	Total (*n* = 96)		Anti-TNF (-) (*N* = 64)		Anti-TNF (+) (*n* = 32)		Anti-TNF(-) vs. (+)
	*r*	*p*	*r*	*p*	*r*	*p*	*p*
**pMayo**	0.442	<0.0001	**0.470**	<0.0001	**0.393**	0.026	**0.660**
**FC**	0.324	0.001	**0.380**	0.002	**0.384**	0.03	**1**
**IL-6**	0.257	0.012	**0.302**	0.015	**0.156**	0.393	**N/A**
**pre-Alb**	−0.474	<0.0001	**−0.394**	0.001	**−0.584**	0.001	**0.271**
**hs-CRP**	0.657	<0.0001	**0.593**	<0.0001	**0.825**	<0.0001	**0.034**
**CRP**	0.681	<0.0001	**0.620**	<0.0001	**0.827**	<0.0001	**0.057**

LRG, leucine-rich alpha-2 glycoprotein; pMayo, partial Mayo score; FC, fecal calprotectin; IL-6, interleukin-6; pre-Alb, prealbumin; hs-CRP, high-sensitivity C-reactive protein; anti-TNF, anti-TNF antibody preparation.

## Data Availability

The data are not publicly available because there is no appropriate site for uploading at present. The data underlying this article will be shared upon reasonable request to the corresponding author.
