# Correction: Tatsumi et al. Biomarkers for Monitoring of Changes in Disease Activity in Ulcerative Colitis. *J. Clin. Med.* 2023, *12*, 7165

**DOI:** 10.3390/jcm13133923

**Published:** 2024-07-04

**Authors:** Yoshihiro Tatsumi, Kazuki Kakimoto, Azusa Hara, Noboru Mizuta, Keijiro Numa, Naohiko Kinoshita, Kei Nakazawa, Ryoji Koshiba, Yuki Hirata, Kazuhiro Ota, Takako Miyazaki, Shiro Nakamura, Kayoko Sakagami, Shoko Arimitsu, Hiroaki Ito, Hiroki Nishikawa

**Affiliations:** 1 2nd Department of Internal Medicine, Osaka Medical and Pharmaceutical University, Takatsuki City 569-8686, Japan; t_cop_doradora@yahoo.co.jp (Y.T.); wakayamakisyu@yahoo.co.jp (A.H.); noboru.mizuta@ompu.ac.jp (N.M.); jeni_like_0902@yahoo.co.jp (K.N.); naohiko.kinoshita@ompu.ac.jp (N.K.); kei.nakazawa@ompu.ac.jp (K.N.); ryouji.koshiba@ompu.ac.jp (R.K.); yuki.hirata.ln@ompu.ac.jp (Y.H.); clash_kaz@yahoo.co.jp (K.O.); takako.miyazaki@ompu.ac.jp (T.M.); saab460@gmail.com (S.N.); hiroki.nishikawa@ompu.ac.jp (H.N.); 2 Kinshukai Infusion Clinic, Osaka-shi 530-0011, Japan; office@kic-clinic.jp (K.S.); aripikari@ares.eonet.ne.jp (S.A.); lovemagicflute@yahoo.co.jp (H.I.)

Text Correction

There was an error in the original publication [1]. **“The authors mentioned ‘anti-IL-6 antibody’ in two places, which was an error and instead should be ‘anti-IL-6 receptor antibody’. The authors have also changed reference 23 accordingly to the following:**

**23. Naka T, Fujimoto M. LRG is a novel inflammatory marker clinically useful for the evaluation of disease activity in rheumatoid arthritis and inflammatory bowel disease. Immunol Med. 2018 Jun;41(2):62–67. doi: 10.1080/13497413.2018.1481582.”** [2].

A correction has been made to ***“Introduction”*, *“Paragraph Number 3”***:

“Another factor that has to be taken into consideration is the interaction between biomarkers. For example, CRP is less likely to be elevated under anti-IL-6 receptor antibody therapy in patients with rheumatoid arthritis since the release of CRP is affected by IL-6; therefore, it is not useful for assessing disease activity [23]. Similarly, LRG is induced by stimuli such as TNFα [12]; therefore, under the administration of anti-TNFα antibody preparations, which are typical biologics for the treatment of refractory UC, the reactivity of LRG may be slowed down with a decrease in TNFα. However, there have been no reports on LRG reactivity in patients with UC receiving anti-TNFα antibody agents.”

A correction has been made to ***“Discussion”*, *“Paragraph Number 3”***:

“Certain biomarkers may be undetectable in patients treated with biological agents. It is known that serum CRP, which is induced by IL-6, is less likely to be elevated in patients with rheumatoid arthritis who are being treated with anti-IL-6 receptor antibody preparations [23]. Similarly, since LRG is induced by stimulation from inflammatory cytokines such as TNFα, IL-1β, and IL-6, this study examined whether LRG is less likely to be detected in patients with UC receiving anti-TNF antibody preparations. No particular change in LRG reactivity was observed in these patients. These results suggest that LRG is a useful marker of disease activity even in patients receiving anti-TNF antibody preparations.”

The authors state that the scientific conclusions are unaffected. This correction was approved by the Academic Editor. The original publication has also been updated.
